# Is trypophobia real?

**DOI:** 10.1192/bjo.2023.621

**Published:** 2024-02-16

**Authors:** Geoff G. Cole

**Affiliations:** Centre for Brain Science, University of Essex, UK

**Keywords:** Anxiety or fear-related disorders, phobias, cognitive neuroscience, trypophobia, aetiology

## Abstract

Approximately 10–18% of the adult population experience some form of anxiety when viewing clusters of small holes. ‘Trypophobia’ has been the subject of much discussion within the peer-reviewed literature, news outlets, health-related websites and social media. However, there is some scepticism surrounding the phenomenon. It is often stated that the condition is not recognised by the American Psychiatric Association, and not listed as a phobia in the DSM-5. It has also been claimed that trypophobia is no more than a particularly successful internet meme. In this editorial, I argue that such criticisms are misplaced. There is, for instance, no list of phobias in the DSM-5; only criteria that determine phobia classification. Using these criteria, as well as personal testimonials, trypophobia is clearly a phobia. Furthermore, the meme hypothesis cannot account for the fact that the phenomenon existed long before the internet.

In 2013, myself and Arnold Wilkins described a phenomenon in which individuals report aversion to clusters of small holes.^1^ Forty-seven peer-reviewed papers have now appeared on ‘trypophobia’, primarily concerned with the critical features of the stimuli that induce the condition and why it occurs (e.g. Yamada and Sasaki^2^). The paper also initiated much discussion on social media and generated hundreds of news articles (e.g. *The Washington Post*, *CNN*, *Daily Mail, Independent, Huffington Post, BBC*). The phenomenon has also been included in many medical and health-related websites. As Ng^3^ stated, trypophobia became a ‘buzzword online’. Thus, the narrative associated with the condition has not come solely from academia. However, many authors from both the peer- and non-peer-reviewed literature, have expressed scepticisms about the phenomenon. Here, I will rebut the central aspects of the discourse: that trypophobia is not a fear or phobia and is no more than a particularly successful internet meme.

## Is trypophobia a fear?

The title of the Cole and Wilkins paper referred to trypophobia as a ‘fear’.^1^ This reflected the fact that the phenomenon had already been given the phobia title (online) and a phobia commonly means to have a ‘fear of’ something. Some authors (e.g. Can et al^4^) have taken this to mean that a fearful response is the central or typical reaction. Although the response of some individuals is best characterised as fear,^5^ most feel anxious as opposed to being scared.^6^ Indeed, trypophobia-inducing stimuli can cause a range of negative reactions, including disgust.^7,8^ The variety of possible emotions is the reason why Cole and Wilkins^1^ described trypophobia as an ‘aversion’ and suggested that, in addition to the psychiatric literature, it could be placed within the context of visual stress in which certain stimuli induce unpleasant effects, as with photosensitive epilepsy.

Trypophobia is evidently an aversion that induces some form of anxiety and discomfort that can be objectively measured (e.g. increased electrodermal activity^9^). Even the fact that trypophobia has been shown to induce disgust^7,8^ does not challenge the idea that phenomenon is an anxiety or aversion; spiders, well-known to induce aversion, also induce disgust.^10^ It is additionally worth noting that the DSM-5^11^ does not separate fear from anxiety when outlining the seven diagnostic criteria for specific phobias; it instead refers to ‘fear or anxiety’ in all seven, and ‘avoidance’ in three. This is, of course, not to state that these responses are physiologically and/or psychologically the same; it simply acknowledges that the differences in fear, anxiety and avoidance may not always be particularly important when assessing phobia.

## Trypophobia and the DSM-5

Many authors have also referred to the fact that trypophobia is not listed in the DSM-5. This has, for some, come to mean that the phenomenon is not formally ‘recognised’ by the American Psychiatric Association (APA).^4,12^ However, this argument misrepresents what the manual states with respect to phobia classification. There is no list of phobias, as there is no list of any other mental disorder. There are ‘codes’, presented in an appendix, which are list-like but, importantly, these are not used to determine whether a behaviour should be considered a phobia. Instead, the DSM-5 presents a series of criteria. It is these that are central to determining whether an aversion reaches the definition of a phobia, not any list. When a phobia is mentioned it is simply provided as an example. Indeed, very few phobias are mentioned but are nonetheless recognised as real phobias. For example, an aversion to cats does not appear anywhere, yet no one would argue that ailurophobia is ‘not recognised’ by the APA.

Importantly, trypophobia can be defined as a specific phobia when using all seven criteria outlined in the DSM-5.

The first criterion is that the aversion occurs in response to a ‘specific object or situation’. Indeed, the inducing stimuli are so specific that they possess relatively greater contrasts at different spatial frequencies compared with non-trypophobic holes.^13,14^ The second criterion is that the threat from the stimuli (e.g. aerated chocolate) is clearly ‘out of proportion to the actual danger posed’. The third criterion is satisfied as, given that trypophobia is induced by a visual stimulus rather than a situation, the response is always ‘immediate’. For the fourth criterion, the many testimonials given on the trypophobia Facebook discussion forum (Trypophobia: Fear of Clusters of Holes; see https://www.facebook.com/groups/3318322299) clearly show that the inducing images are ‘actively avoided’. Indeed, one of the moderating rules of this forum is that trypophobic images must not be posted. Such active avoidance is also supported by the peer-reviewed literature; Robakis^5^ reported that one trypophobic individual was not able to drive because of the light-emitting diode clusters in traffic lights. These testimonials also show that the condition fulfils the fifth criterion of ‘typically lasting for 6 months or more’, with sufferers often stating that their trypophobia began in childhood. Furthermore, in a survey of 195 users of the trypophobia Facebook discussion forum, Vlok-Barnard and Stein^6^ found that the condition had a mean duration of 18 years and a range that went up to 60 years. The sixth criterion in the DSM-5 also states that the phobia must not be ‘better explained by the symptoms of another mental disorder’ that also include fear, anxiety and avoidance. Four peer-reviewed article have now shown that trypophobia is weakly,^1^ if at all, associated with general anxiety^15,16^ and general mental health outcomes.^17^ Finally, for the seventh criterion, trypophobic images can create ‘significant distress or impairment in social, occupational, or other important areas of functioning’. The Vlok-Barnard and Stein survey also showed that 15.4% of respondents scored at least five on the work or school subscale of the Sheehan Disability Scale (SDS).^18^ Such a score classifies an individual as having ‘significant impairment’ in the relevant activity. With specific reference to ‘distress’, the survey additionally found that 25.8% of respondents reported ‘a high level of psychological distress’ and a further 24.7% reported ‘a very high level’.

This last DSM-5 criterion also shows how comparable trypophobia is to other specific phobias. For example, in a worldwide sample of over 7000 participants with specific phobia, Wardenaar et al^19^ found that only 10.3% scored at least seven (‘severe impairment’) on the work subscale of the SDS. As Becker et al^20^ noted, ‘Specific phobias are often considered less impairing than other disorders, since the feared object or situation is circumscribed and therefore its avoidance is much easier than, for example, in social phobia or agoraphobia’.

Trypophobia also reaches the definition of a specific phobia as outlined by the alternative ICD-11,^21^ published by the World Health Organization. This is to be expected, given the large overlap of the two instruments with respect to the criteria for a specific phobia. Six ‘essential’ or ‘required features’ are described. The ‘fear, anxiety, or avoidance’: consistently occurs upon exposure, is out of proportion to the actual danger posed, lasts for at least several months, is not better accounted for by another mental disorder, impairs functioning in a range of situations (e.g. educational, occupational) and is actively avoided. Note that the ICD-11 also allows for odd or unusual phobias, which could include trypophobia.

## Is trypophobia no more than a successful internet meme?

The issue of ‘recognition’ is related to perhaps the most serious criticism: that trypophobia does not exist at all. For example, Can et al^4^ suggest that individuals only ‘claim’ to have trypophobia, and Smith,^22^ writing in *Medical News Today*, asks ‘Is trypophobia real?’. To challenge the notion that people report negative reactions to trypophobic stimuli is an odd position to take. Scepticism of the basic phenomenon is also reflected in the phrase that a person can ‘have a phobia to anything’.^23^ Although classical conditioning can result in phobia to any stimulus, opponents have to explain why 10–18% of the adult population^17,24^ are averse to a very diverse range of trypophobic stimuli that all have a particular spectral property in common: relatively high contrasts at a range of spatial frequencies, which is a property that individuals would not be conscious of. Thus, it is difficult (for opponents) to explain why manipulations in luminance contrast^14^ and phase spectrum influence responses.^15^ This cannot be easily explained through conditioning. Furthermore, trypophobic stimuli have been shown to induce other very specific effects. For example, they subtly influence eye movement trajectories in a way that does not occur with control images.^25^

Although the present author rejects criticisms concerning response (i.e. fear, response or avoidance) and DSM-5 classification, it is possible that some people have ‘caught’ trypophobia via its ubiquitous online presence, or as Oelze^26^ states, ‘a fear made worse by the internet’. It is certainly the case that many people first came across trypophobia via a number of widely circulated images in which holes were digitally incorporated onto human skin. However, even if the internet meme theory is true this would be no different to many other phobias. That is, a condition passed on via social influence in which a person becomes aware of an aversion experienced by, for example, a family member. There is also the acquisition of knowledge concerning societal representation and view of certain objects (e.g. snakes, cockroaches). The present author will also note that many of the 100 or so individuals with trypophobia he has had contact with in the past decade stated that they remember having the condition before the internet existed. Typically, these individuals have been aged ≥50 years and recall first seeing the inducing stimuli as children in the 1970s and 1980s. This clearly refutes the notion that trypophobia is solely an internet-induced phenomenon.

## Future work and conclusions

A major challenge is to identify aetiology. In contrast to the social learning theories are evolutionary accounts. For instance, the spectral composition of trypophobic stimuli, in terms of contrasts and spatial frequency, is particularly unique and shared with the aposematic patterns typically present on the skin of poisonous animals.^1^ Humans may therefore have evolved to be particularly sensitive to this feature. The condition may also be related to sensitivity toward skin disease and pathology, which often present as clusters of circles.^2,7^ Indeed, incorporating hole clusters onto (images of) human skin significantly increases the degree to which the images are judged to be averse.^16^ There is also the issue of how reactions to trypophobia-inducing stimuli are best characterised. Evidence so far suggests that the most common response is itchiness, goosebumps and nausea.^6^ Disgust is also common.^7,8^ Future large-scale studies also need to determine specific prevalence. The largest study conducted so far, by Cole et al^24^ (*N* = 2558, 47% men, UK citizens, a range of socioeconomic backgrounds and educational history, relatively even distribution of participants across six age categories ranging from 18 to ≥65 years), shows that 9.7% of respondents reached the threshold for being defined as trypophobic, as determined by the commonly used Trypophobia Questionnaire.^13^ Furthermore, although 57% of respondents indicated that they felt no anxiety at all when viewing the two trypophobic images presented as part of the scale, 33% responded ‘slightly’ or ‘moderately’ on at least one of the 17 items that asks observers to consider the degree to which a statement is appropriate to them (e.g. ‘Feel aversion, disgust or repulsion’, ‘Have trouble breathing’). This suggests that trypophobia is a matter of degree that can include a milder form. Although the condition satisfies the criteria for being clinically significant (e.g. persists for at least 6 months), this study did not determine whether positive participants met the criteria for specific phobia. Thus, some individuals may experience aversion to hole clusters without meeting the DSM-5 criteria. A further large-scale (*N* = 2065) assessment of trypophobia places the prevalence figure at 17.6%.^17^ However, this was conducted on individuals aged 15–24 years, and it is known that instances of phobia decreases as age increases,^27^ including trypophobia.^24^ Taken together,^17,24^ prevalence of trypophobia is between 10 and 18%. This fits well with the first estimate of around 16%.^1^ It is also important for future studies to determine the comorbidities associated with trypophobia. For example, Wong et al^17^ noted that, ‘As young people are often less likely to seek professional help for mental health needs, incorporating measures of trypophobia in future routine screening may improve both youth engagement and possible earlier detection of young people at greater psychiatric risk’. In this context, Wong et al found that symptoms of depression and trypophobia were significantly, albeit weakly, associated. Finally, there is the issue of potential treatments. Although there is not yet firm evidence on this issue, it may be the case that interventions known to help with the treatment of other specific phobias will be effective.

**Fig. 1:**
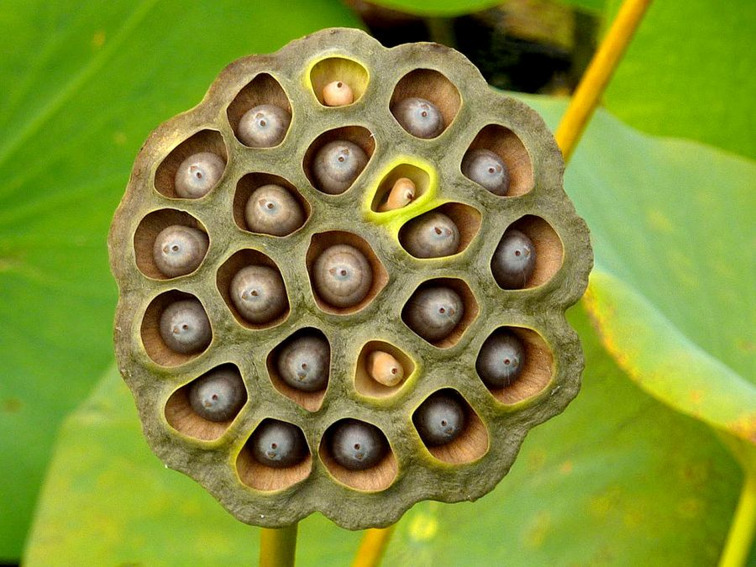
Image of a lotus seed pod.

In sum, clusters of holes induce various forms of anxiety that can be measured both psychologically and physiologically.^28^ As with most other phobias, the phenomenon is not mentioned or ‘listed’ in the DSM-5. Trypophobia does, however, reach the definition of a specific phobia; a phobia that, furthermore, is not uncommon. Indeed, the present author is currently examining the possibility that everyone is sensitive to trypophobic stimuli – although they may not feel anxious, most people can appreciate that there is something not quite right about the lotus seed pod (see Fig. 1).
